# Identification of a novel nine‐SnoRNA signature with potential prognostic and therapeutic value in ovarian cancer

**DOI:** 10.1002/cam4.4598

**Published:** 2022-02-21

**Authors:** Wenjing Zhu, Tao Zhang, Shaohong Luan, Qingnuan Kong, Wenmin Hu, Xin Zou, Feibo Zheng, Wei Han

**Affiliations:** ^1^ Clinical Research Center Qingdao Municipal Hospital, Qingdao University Qingdao China; ^2^ Department of Respiratory and Critical Care Medicine, Qingdao Municipal Hospital, School of Medicine Qingdao University Qingdao Shandong China; ^3^ Respiratory Disease Key Laboratory of Qingdao Qingdao Municipal Hospital Qingdao China; ^4^ Department of Gynecology, Qingdao Municipal Hospital, School of Medicine Qingdao University Qingdao Shandong China; ^5^ Department of Pathology Qingdao Municipal Hospital Qingdao Shandong Province China; ^6^ School of Medicine and Pharmacy Ocean University of China Qingdao China; ^7^ Department of Pathology Qingdao Municipal Hospital Dalian Medical University Dalian China; ^8^ Department of Nuclear Medicine Qingdao Municipal Hospital, Qingdao University Qingdao China

**Keywords:** biomarker, ovarian cancer, prognosis, SnoRNA

## Abstract

**Background:**

Increasing evidence has been confirmed that small nucleolar RNAs (SnoRNAs) play critical roles in tumorigenesis and exhibit prognostic value in clinical practice. However, there is short of systematic research on SnoRNAs in ovarian cancer (OV).

**Material/Methods:**

379 OV patients with RNA‐Seq and clinical parameters from TCGA database and 5 paired clinical OV tissues were embedded in our study. Cox regression analysis was used to identify prognostic SnoRNAs and construct prediction model. SNORic database was adopted to examine the copy number variation of SnoRNAs. ROC curves and KM plot curves were applied to validate the prognostic model. Besides, the model was validated in 5 paired clinical tissues by real‐time PCR, H&E staining and immunohistochemistry.

**Results:**

A prognostic model was constructed on the basis of SnoRNAs in OV patients. Patients with higher RiskScore had poor clinicopathological parameters, including higher age, larger tumor size, advanced stage and with tumor status. KM plot analysis confirmed that patients with higher RiskScore had poorer prognosis in subgroup of age, tumor size, and stage. 7 of 9 SnoRNAs in the prognostic model had positive correlation with their host genes. Moreover, 5 of 9 SnoRNAs in the prognostic model correlated with their CNVs, and SNORD105B had the strongest correction with its CNVs. ROC curve showed that the RiskScore had excellent specificity and accuracy. Further, results of H&E staining and immunohistochemistry of Ki67, P53 and P16 confirmed that patients with higher RiskScore are more malignant.

**Conclusions:**

In summary, we identified a nine‐SnoRNAs signature as an independent indicator to predict prognosis of OV, providing a prospective prognostic biomarker and potential therapeutic targets for ovarian cancer.

## Graphical abstract

Our study established a nine‐snoRNA signature as an independent prognosis indicator for OV, and it may provide potential therapeutic targets for ovarian cancer.

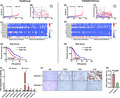

## 1 BACKGROUND

Ovarian cancer is the leading cause of death in gynecological cancer, since patients with early stage ovarian cancer do not have symptoms of discomfort and more than half of patients have reached advanced stage (stage III or IV).^1^ Although the incidence rate of other gynecological cancers such as endometrial cancer is high, the mortality rate of ovarian cancer is still the highest.^2^ More than 75% of ovarian cancers are diagnosed at advanced or metastatic stage.^3^ Besides, the treatments for ovarian cancer were limited because of the recurrence and resistance in patients diagnosed with high‐grade serous ovarian cancer.^4^ Despite initially responding to radiotherapy and chemotherapy treatment, recurrence is likely to occur within a median of 16 months for patients who present with advanced stage disease.^5^ At present, identifying and discovering effective biomarkers and realizing molecular targeted therapy are considered to be an effective treatment for ovarian cancer.^6^ Consequently, finding effective therapeutic target molecules for ovarian cancer is an urgent problem to be solved.

Small nucleolar RNAs (SnoRNAs) are a class of non‐coding RNAs with 60–300 nt, and mainly divided into two classes: C/D box SnoRNAs and H/ACA box SnoRNAs.^7^ Traditionally, they act as the role of modifying 2′‐O‐ribose methylation and pseudouridylation of ribosomal RNAs (rRNAs), respectively.^7^ Emerging evidence has demonstrated that small nucleolar RNAs (SnoRNAs) play significant roles in tumorigenesis.^8^ Such as, SnoRNA U3, a box of C/D RNA, could be processed to smaller RNAs just as miRNA and perform the function of miRNA in cancer.^9^ Moreover, SnoRNAs had been reported to play a critical determinant of leukemic stem cell activity, and disruption in the level of H/ACA SnoRNAs in stem cells impairs pluripotency.^10^, ^11^ Further, other research suggested that SnoRNAs participated in the regulation of mRNA abundance, alternative splicing, and metabolic and oxidative stress.^12^

Recent study showed that SnoRNAs could act as diagnostic markers, prognostic markers and therapeutic targets in various cancers.^13^ The number of dysregulated SnoRNAs in ovarian cancer is up to 462^8^; however, there is no study on SnoRNAs had been conducted in ovarian cancer.

Thus, we screened out the prognostic SnoRNAs in ovarian cancer and constructed a risk model to predict the prognosis for ovarian cancer patients. This may provide new ideas and targets for the clinical treatment of ovarian cancer.

## 2 METHODS

### 2.1 Data sets

The data of patients with ovarian cancer in TCGA, including RNA‐Seq data and clinical data, were downloaded by the GDC data portal: https://portal.gdc.cancer.gov/. The detailed clinical pathological parameters of patients with ovarian cancer, including age, subdivision, lymphatic invasion, grade, race, stage, tumor size and venous invasion of ovarian cancer, were listed in Table 1.

**TABLE 1:** Clinical pathological parameters of ovarian cancer patients in TCGA database

Clinical pathological parameters	*N*	%
Age		
≤60	206	54.9
>60	169	45.1
Subdivision		
Left or right	50	28.6
Bilateral	125	71.4
Lymphatic invasion		
No	20	30.3
Yes	46	69.7
Grade		
G1 + G2	21	11.5
G3 + G4	162	88.5
Race		
Asian	4	2.2
Black or African American	15	8.2
White	163	89.6
Stage		
Stage1 + 2	12	6.5
Stage3 + 4	173	93.5
Tumor size		
No Macroscopic disease	36	22.1
≤20 mm	94	57.7
>20 mm	33	20.2
Venous invasion		
No	19	36.2
Yes	39	63.8

### 2.2 Patients and clinical specimens

We recruited 5 pairs of matched ovarian cancer tissues and normal tissues from Chinese Institution. Among them, three cases were diagnosed as high‐grade serous carcinoma with pleomorphic nuclei, high N/C ratio and active mitosis. One case was diagnosed as low‐grade serous carcinoma composed of small cellular nests containing multiple psammoma bodies, uniform nuclei with mild to moderate atypia. One case was diagnosed as endometrioid adenocarcinoma which displayed tubular pattern and nests.

These tissue samples and corresponding clinical pathology data were collected from Qingdao Municipal Hospital. This study was approved by Institutional Review Board of Qingdao Municipal Hospital. The number of the approval of this study by the ethical committee is No.018. And the approval document was approved on September, 2021.

### 2.3 RNA isolation and quantitative real‐time PCR (qRT‐PCR)

For tissue RNA isolation, 1 ml AG RNAex Pro Reagent (Accurate Biotechnology Co.) was added to 50 mg of tissue and total RNA samples were extracted according to the manufacturer's instructions. Purified RNA was quantified resort to NanoVue (GE Healthcare Life Sciences). cDNAs were synthesized from total RNAs by using RT reagent Kit (Takara Co., LTD) and ReverTra Ace qPCR RT Kit (Toyobo Co., LTD).^14^

qRT‐PCR of U6, SNORA11B, SNORA36C, SNORA58, SNORA70J, SNORA75B, SNORD105B, SNORD126, SNORD3C and SNORD89 was performed with the SYBR qPCR Mix (Toyobo Co., LTD). 10 μl reaction system was adopted according to the manufacturer's instructions and amplified for 40 cycles. The expression levels were normalized by U6. Relative expression was calculated using the method of 2^−ΔΔCt^ and the expression levels of SnoRNAs were calculated using the 2^−ΔCt^ method.^15^ Primer names and primer sequences are listed in the following tables (Tables 2 and 3). Quantification of U6 was performed with a stem‐loop real time PCR miRNA kit (Ribobio Co., LTD).

**TABLE 2:** The forward and reverse primer sequence of the SnoRNAs

Primer name	Primer sequence
SNORA58 Forward	TTGCCTGACTGTGCTCATGTC
SNORA58 Reverse	GGGAAATGTTTAGAGTCCTGCAAT
SNORD89 Forward	CAAGAAAAGGCCGAATTGCA
SNORD89 Reverse	TTCGCTTCAGGATATTTTGTCATC
SNORA70J Forward	GCCAATTAAGCCGACTGAGTTC
SNORA70J Reverse	ACAGGCTGCATATACTACCAAGGAA
SNORD3C Forward	CGAGGAAGAGAGGTAGCGTTTTC
SNORD3C Reverse	CGGAGAGAAGAACGATCATCAA
SNORA75B Forward	AGAAGAGAGAATTCACAGAACTAGCG
SNORA75B Reverse	AGTGCAGGGTCCGAGGTATT
SNORD126 Forward	GCCATGATGAAATGCATGTTAAGTCC
SNORD126 Reverse	AGTGCAGGGTCCGAGGTATT
SNORD105B Forward	GACAGCACTTCTGCTGAGACG
SNORD105B Reverse	AGTGCAGGGTCCGAGGTATT
SNORA11B Forward	CCTCCTCTGTTTACAACACACCCA
SNORA11B Reverse	AGTGCAGGGTCCGAGGTATT
SNORA36C Forward	GGCAGCTTCCCTGTTCTGTT
SNORA36C Reverse	AGTGCAGGGTCCGAGGTATT

**TABLE 3:** The RT‐primer sequence of the SnoRNAs

Primer name	Primer sequence
SNORA75B	GTCGTATCCAGTGCAGGGTCCGAGGTATTCGCACTGGATACGACGAATGT
SNORD126	GTCGTATCCAGTGCAGGGTCCGAGGTATTCGCACTGGATACGACCCTAGC
SNORD105B	GTCGTATCCAGTGCAGGGTCCGAGGTATTCGCACTGGATACGACCCTTCC
SNORA11B	GTCGTATCCAGTGCAGGGTCCGAGGTATTCGCACTGGATACGACTGTGTA
SNORA36C	GTCGTATCCAGTGCAGGGTCCGAGGTATTCGCACTGGATACGACTTTGTA

Primers of SNORA58, SNORD89, SNORA70J and SNORD3C synthesized by probe method. The other primers were synthesized by stem‐roop method from Sangon Biotech Company, and the RT‐Primers as Table 3.

### 2.4 Clonogenic assay

Ovarian cancer cells (1000 cells/well) transfected with snoRD89 or snoRD126 overexpression (OE) plasmids were placed in 6‐well plates and maintained in medium containing 15% FBS. After 14 days, the cells were fixed and stained by crystal violet.

### 2.5 Statistical analysis

Univariate Cox regression analysis was used to screen prognostic genes with *p* values of <0.05. Then, multivariate cox regression analysis was adopted to establish a prognostic risk score model. According to the prognostic risk score model, each ovarian cancer patient had an unique RiskScore, and the RiskScore was calculated by the risk score formula = *β*1*expression of gene 1 + *β*2*expression of gene 2 + *β*3*expression of gene 3 + …. + *β*n*expression of gene N. Paired *t* test were used to compare the expression of genes in ovarian cancer tissues versus normal tissues. According to the median RiskScore, ovarian cancer patients were divided into high‐risk group and low‐risk group. Receiver operating characteristic (ROC) curves and KM plot curves were used to validate the prognostic model. The Log‐rank (Mantel–Cox) test was used for survival analysis by GraphPad Prism 7.0. Differences were considered statistically significant when the *p*‐value was <0.05.

## 3 RESULTS

### 3.1 Construction of prognostic model for ovarian cancer patients

432 SnoRNAs were detected in ovarian cancer patients from TCGA. Univariate Cox survival analysis showed that 14 SnoRNAs had an effect on the prognosis of ovarian cancer patients (Table 4). Multivariate Cox survival analysis was adopted to conduct prognostic model, and finally 9 SnoRNAs were screened out. RiskScore = −0.7390*SNORA11B + 0.8479*SNORA36C − 0.6813*SNORA58 + 2.2898*SNORA70J + 2.4864*SNORA75B − 0.4467*SNORD105B + 1.1156*SNORD126 + 3.3939*SNORD3C + 0.4938*SNORD89.

**TABLE 4:** Univariate Cox survival analysis showed that 14 SnoRNAs had an effect on the prognosis of ovarian cancer patients

Gene	HR	*z*	*p* value
SNORD126	3.03664	3.519229	0.000433
SNORA70J	9.488882	3.448622	0.000563
SNORD3C	23.99664	3.174154	0.001503
SNORA75B	65.38938	3.010749	0.002606
SNORA58	0.626393	−2.47562	0.0133
SNORA11B	0.501669	−2.23471	0.025436
SNORA36C	2.031193	2.141889	0.032202
SNORD105B	0.62715	−2.05778	0.039611
SNORD89	1.271647	2.000204	0.045478
SNORD116‐25	2.672161	1.99728	0.045795
SNORA30B	10.92123	1.988977	0.046704
SNORD116‐2	1.406725	1.98367	0.047293
SNORD105	0.61692	−1.96876	0.04898

**FIGURE 1:**
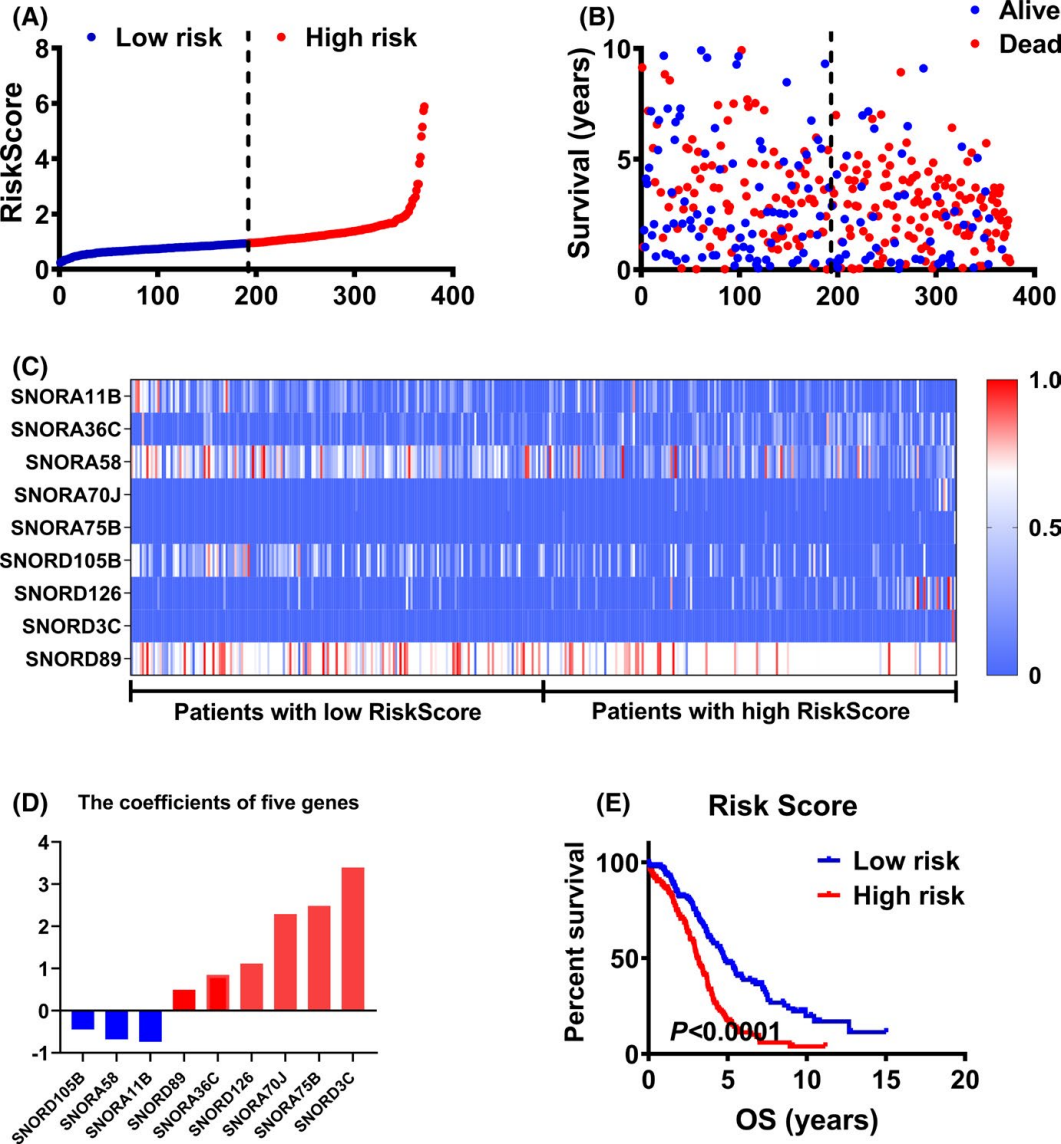
Construction of the prognostic model for ovarian cancer. (A) the RiskScore of ovarian cancer patients in TCGA database was ranked from low to high; (B) scatter heat map was drawn including RiskScore, survival time and vital status of ovarian cancer patients; (C) the expression heat map of SnoRNAs in patients with low RiskScore and high RiskScore; (D) the coefficient of SnoRNAs in the RiskScore formula (prognostic model); (E) K–M plot survival curve of ovarian cancer patients with low RiskScore versus high RiskScore

According to the RiskScore formula, all ovarian cancer patients had a unique RiskScore, and we ranked the patients according to their RiskScore (Figure 1A). Scatter plot was used to analyze the RiskScore, survival time and survival state of ovarian cancer patients, and we found that patients with higher RiskScore had lower survival time and more deaths than that with lower RiskScore (Figure 1B). The expression of SnoRNAs in the prognostic model was compared in patients with low RiskScore versus high RiskScore (Figure 1C). In the RiskScore model, three SnoRNAs had negative coefficient, and among of them, snoRD3C had the largest weight coefficient in the prognostic model (Table 5 and Figure 1D). Moreover, we compared the survival time of ovarian cancer patients with high RiskScore to low RiskScore. Patients with high RiskScore had poorer prognosis than those with low RiskScore (Figure 1E).

**TABLE 5:** The results of multivariate Cox survival analysis

Gene	Coef	Exp (coef)	SE (coef)	z	*p* value
SNORA11B	−0.7390	0.4776	0.3184	−2.321	0.020275
SNORA36C	0.8479	2.3347	0.3426	2.475	0.013326
SNORA58	−0.6813	0.5060	0.2057	−3.312	0.000925
SNORA70J	2.2898	9.8732	0.6960	3.290	0.001002
SNORA75B	2.4864	12.0180	1.3625	1.825	0.068017
SNORD105B	−0.4467	0.6397	0.2290	−1.951	0.051102
SNORD126	1.1156	3.0514	0.3231	3.453	0.000554
SNORD3C	3.3939	29.7825	1.0515	3.228	0.001248
SNORD89	0.4938	1.6385	0.1400	3.528	0.000419

### 3.2 Patients with high riskscore had poor clinicopathological stratification

In order to determine whether the RiskScore is related to the clinicopathological parameters of ovarian cancer patients, we analyzed the level of the RiskScore in different subgroups of the clinicopathological parameters. Results showed that patients with higher age, larger tumorsize, advanced stage and with tumor status had higher RiskScore versus the other subgroup (*p* < 0.05, Figures S1A and S2B,D,E). Although there is no statistic statistical significance, patients with lymphatic invasion had higher RiskScore (Figure S1C).

### 3.3 SnoRNAs in the prognostic model co‐expressed with their host genes

**FIGURE 2:**
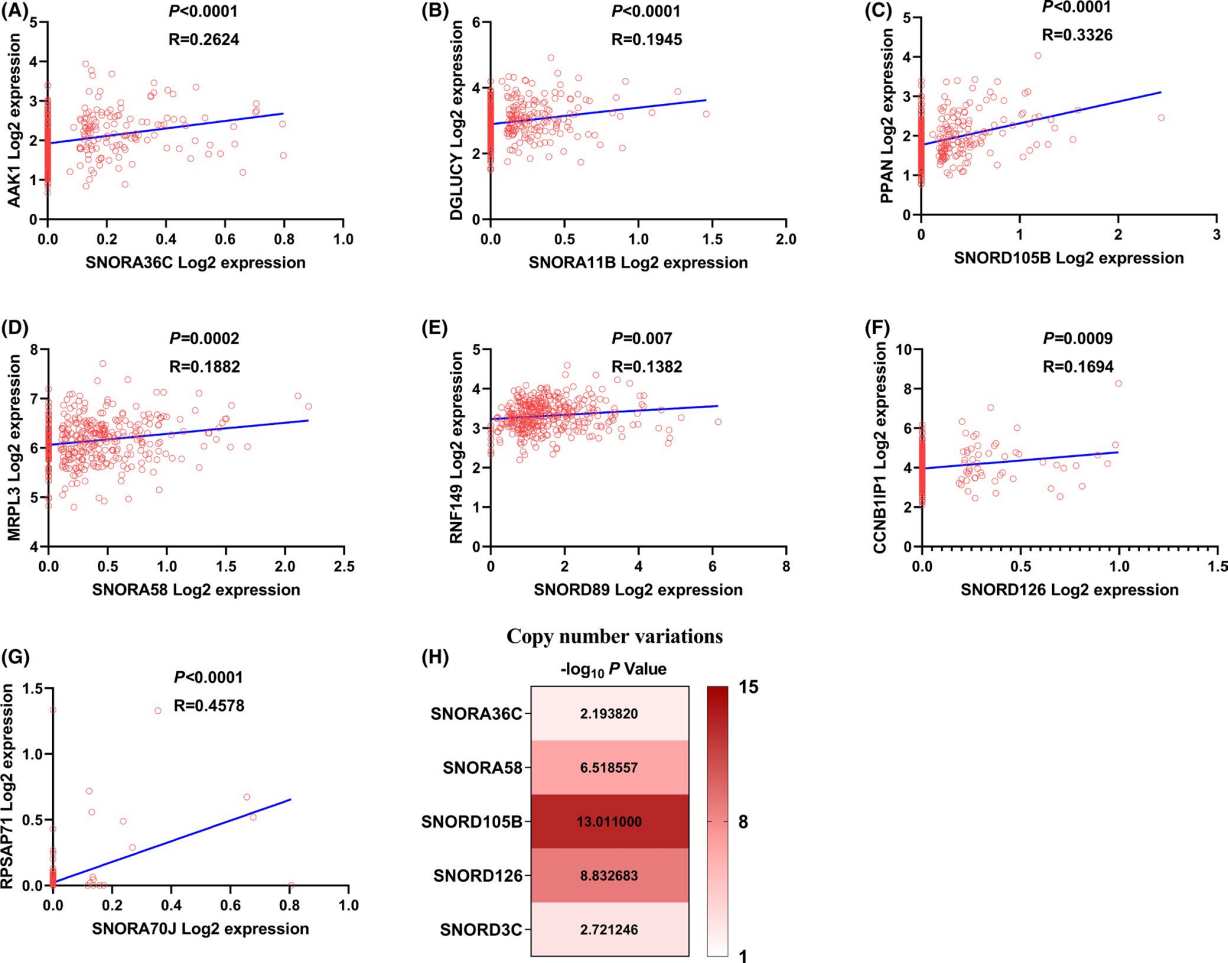
Seven of nine SnoRNAs in the prognostic model co‐expressed with their host genes. (A) SNORA36C; (B) SNORA11B; (C) SNORD105B; (D) SNORA58; (E) SNORD89; (F) SNORD126; (G) SNORA70J; (H) five SnoRNAs in the prognostic model correlated with their CNVs

SnoRNAs exist in the introns of mRNA or LncRNA, and some of them co‐expressed with their host genes.^16^ We compared the correlation of SnoRNAs and their host genes in ovarian cancer tissues. Results showed that 7 SnoRNAs in the prognostic model had positive correlation with their host genes (Figure 2A–G). Among them, the expression abundance of snoRA70J, alike its host gene, is very low in ovarian cancer tissues (Figure 2G). Moreover, copy number variation (CNV) is a key regulator of gene expression, and some SnoRNAs were significantly associated with their CNVs in various cancers.^17^ SNORic database (http://bioin fo.life.hust.edu.cn/SNORic) was used to examine the correlation between SnoRNAs and their copy number variation (CNV). 5 of 9 SnoRNAs in the prognostic model correlated with their CNVs, and SNORD105B had the strongest correction with its CNVs (Figure 2H).

### 3.4 RiskScore is an independent prognostic factor for ovarian cancer patients

**FIGURE 3:**
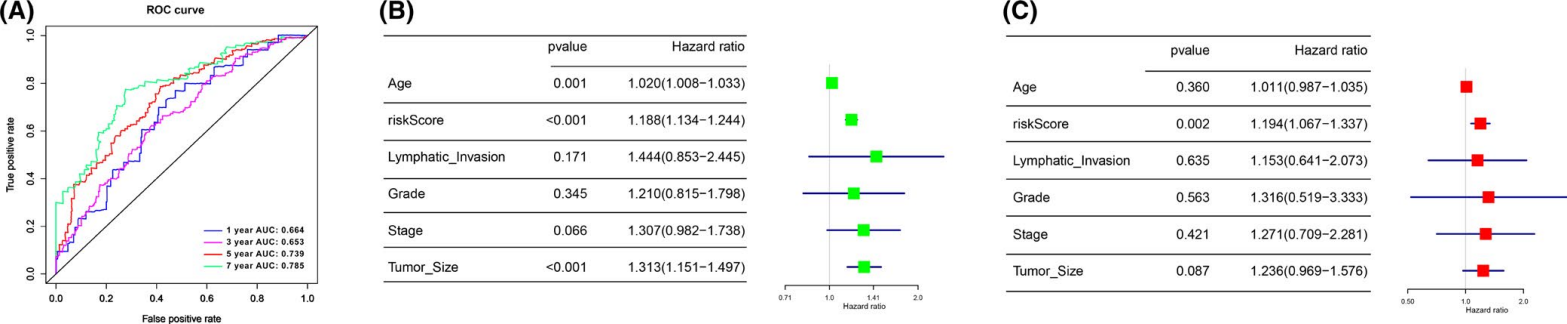
RiskScore derived from the prognostic model is an independent prognostic factors for ovarian cancer patients. (A) ROC analysis of the sensitivity and specificity of 1‐year, 3‐year and 5‐year survival prediction by the nine‐snoRNA RiskScore; (B, C) univariate and multivariate Cox survival analysis were conducted to analyze factors that had effect on the prognosis of ovarian cancer patients

In order to validate the accuracy and specificity of the RiskScore derived from the prognostic model we constructed, ROC curve was adopted. Results showed that the prognostic accuracy of the signature was 0.664, 0.653, 0.739 and 0.785 for 1, 3, 5, and 7 years in entire series which increased with time prolonging (Figure 3A). Hence, the RiskScore has the greatest accuracy and specificity when predicting for 7 years.

Further, univariate and multivariate Cox survival analysis were conducted to analyze factors that had effect on the prognosis of ovarian cancer patients. Univariate Cox survival analysis showed that age, RiskScore, Tumor Size were the dependent prognostic factors in ovarian cancer patients (Figure 3B). Multivariate Cox survival analysis showed that RiskScore was the independent prognostic factor in ovarian cancer patients (Figure 3C). Taken together, the RiskScore from nine SnoRNA signature is a potentially helpful biomarker for predicting the prognosis for ovarian cancer patients.

### 3.5 RiskScore act as a good indicator for prognosis in different clinical subgroups

**FIGURE 4:**
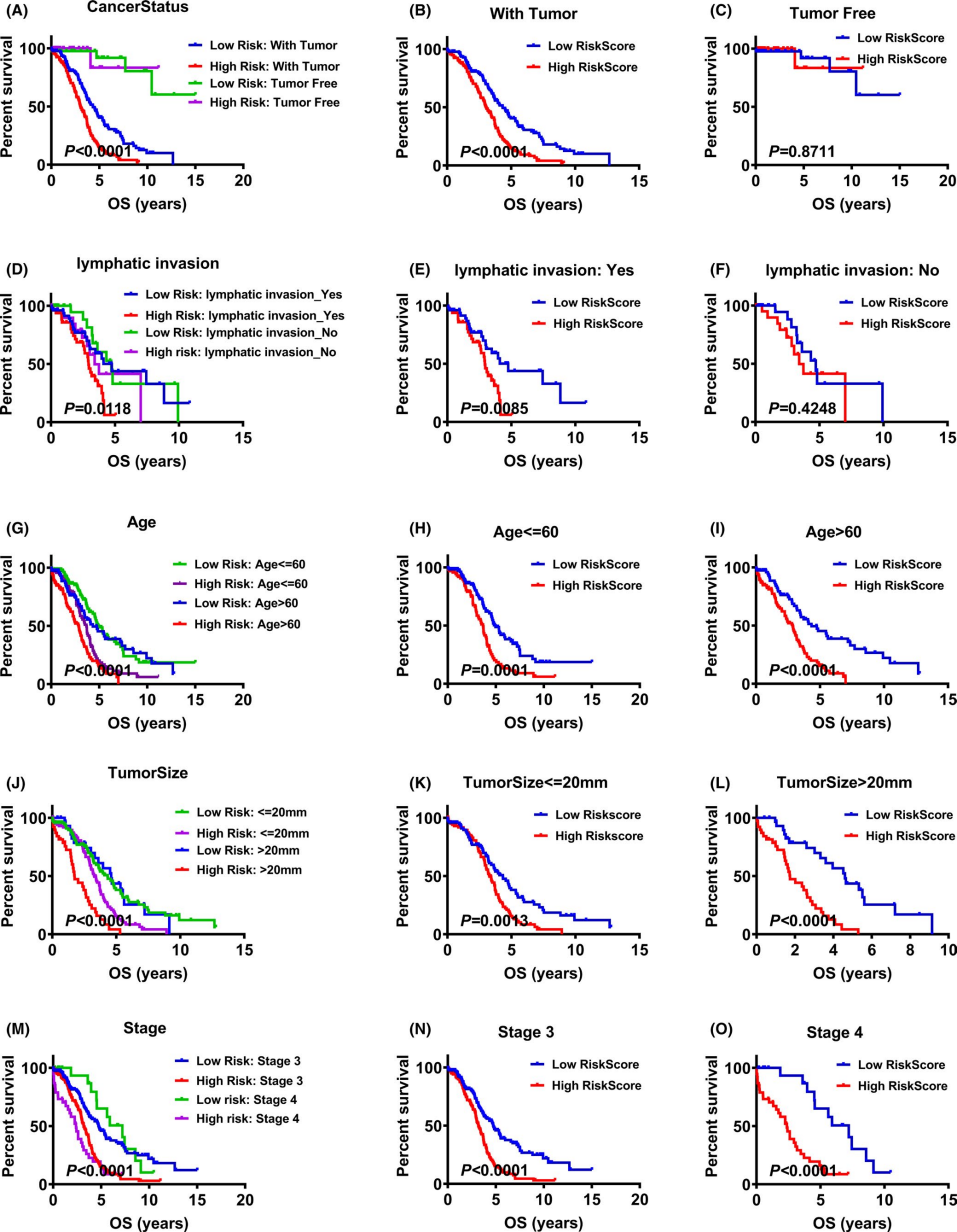
KM plot curves of RiskScore in different clinical subgroups. (A) KM plot curves of RiskScore in patients with different cancer status (with tumor and tumor free); (B, C) KM plot curves of RiskScore in patients with tumor and tumor free; (D) KM plot analysis of RiskScore in patients with different lymphatic invasion status (yes and no); (E, F) KM plot curves of RiskScore in patients with lymphatic invasion and no lymphatic invasion; (G) KM plot analysis of RiskScore in patients with different age (<=60 and >60); (H, I) KM plot curves of RiskScore in patients with age <=60 and age >60; (J) KM plot analysis of RiskScore in patients with different TumorSize (<=20 mm and >20 mm); (K, L) KM plot curves of RiskScore in patients with TumorSize <=20 mm and TumorSize >20 mm; (M) KM plot analysis of RiskScore in patients with different stage (3 and 4); (N, O) KM plot curves of RiskScore in patients with stage 3 and stage 4

In order to confirm whether the RiskScore in different clinical subgroups can be a good indicator for prognosis, KM plot analysis was used. Cancer status have an effect on the prognosis of patients, hence, we first stratified patients into, with tumor and tumor free, two groups. Then, each group was divided into high‐ and low‐risk groups according to their median RiskScore. As results shown in Figure 4A, patients in high‐risk group had significantly shorter OS than those in low‐risk group in with tumor group (Figure 4A,B). In addition, patients with high RiskScore in lymphatic invasion group had poorer prognosis (Figure 4D,E). However, RiskScore cannot discriminate tumor free group and no lymphatic invasion group (Figure 4C,F). These results showed that RiskScore can predict the prognosis of patients with tumor and lymphatic invasion better than the other relevant group.

Besides, age, tumor size and stage are critical clinicopathological parameter affecting the prognosis of ovarian cancer patients.^18^ Therefore, we divided the patients according these clinicopathological parameters, and then compared the prognosis of high RiskScore group to low RiskScore group. As results shown in Figure 4G–I, patients in high RiskScore group had significantly shorter OS than those in low RiskScore group no matter in age ≤60 or age >60 group (*p* < 0.05, Figure 4G–I). Alike, the results in different tumor size group and stage group, patients with high RiskScore had poorer prognosis versus to patients with low RiskScore (*p* < 0.05, Figure 4J–O).

### 3.6 Validation of the prognostic model derived from SnoRNAs

**FIGURE 5:**
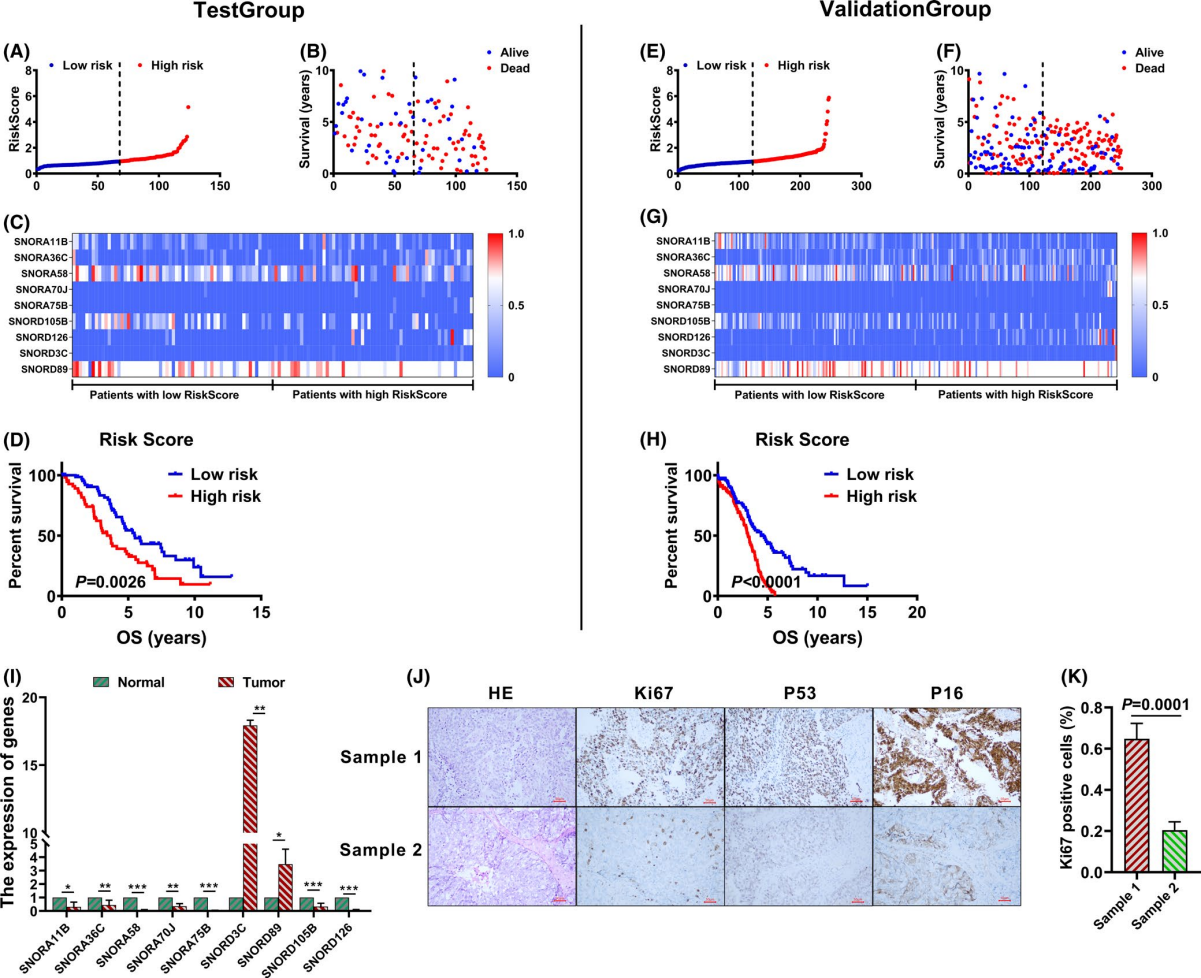
Validation of the RiskScore derived from nine‐snoRNA prognostic model. (A and E) the RiskScore of patients in test group and validation group ranked from low to high; (B and F) scatter heat map was drawn including RiskScore, survival time and vital status of ovarian cancer patients in test group and validation group; (C and G) the expression heat map of SnoRNAs of patients with low RiskScore and high RiskScore in test group and validation group; (D and H) K–M plot survival curve of ovarian cancer patients with low RiskScore versus high RiskScore in test group and validation group; (I) the expression of SnoRNAs in clinical tumor tissues versus normal tissues; (J) HE staining of clinical tumor tissues, and immunohistochemistry was used to detect the expression of Ki67, P53 and P16 in ovarian cancer tissues; (K) the percentage of Ki67 positive cells in ovarian cancer sample 1 versus sample 2. *p < 0.05, **p < 0.01, ***p < 0.001

To further validate the prognostic value of the RiskScore derived from nine‐SnoRNAs for ovarian cancer, we randomly divided the patients into two groups. 125 and 250 cases included in the test group and validation group. Ovarian cancer patients of each group were ranked and divided into two groups according to the median RiskScore (Figure 5A,E). Scatter plot show that patients with high RiskScore had shorter overall survival and higher deaths (Figure 5B,F). Moreover, the expression of SnoRNAs in the prognostic model was compared in test group and validation group (Figure 5C,G). And, KM plot analysis showed that patients with high RiskScore in test group and validation group had poorer prognosis versus to patients with low RiskScore (Figure 5D,H).

Further, we recruited 5 paired clinical tissues to verify our research. Of them, three cases were diagnosed as high‐grade serous carcinoma with pleomorphic nuclei, high N/C ratio and active mitosis. Two cases was diagnosed as low‐grade serous carcinoma composed of small cellular nests containing multiple psammoma bodies, uniform nuclei with mild to moderate atypia (Table 6).

**TABLE 6:** Clinical pathological parameters of 5 paired ovarian cancer from clinical patients

Sample	RiskScore	Histological type	P53 genotype
1	46.47163	High‐grade serous carcinoma	non‐sense mutation
2	2.449066	Low‐grade serous carcinoma	wild type
3	10.048	Low‐grade serous carcinoma	wild type
4	21.526	High‐grade serous carcinoma	missense mutation
5	39.456	High‐grade serous carcinoma	missense mutation

We tested the expression of SnoRNAs in the 5 paired clinical tissues, 7 of 9 SnoRNAs in the prognostic model, including SNORA11B, SNORA36C, SNORA58, SNORA70J, SNORA75B, SNORD3C, SNORD89, SNORD105B and SNORD126, down regulated in tumor tissues versus their paired normal tissues (Figure 5I). In addition, we performed H&E staining on tumor tissues, and immunohistochemistry was used to detect the expression of Ki67, P53 and P16 in tumor tissues. The multiplication capacity of tumor tissues was indicated through Ki‐67 expression measured by immunohistochemistry assays. Among the five clinical patients, sample 1 and sample 2 have the highest and lowest risk values, respectively (Table 6). And, results of H&E staining and immunohistochemistry in sample 1 and sample 2 are exhibited in the Figure 5J. P53 protein was mutated in high‐grade serous carcinoma with non‐sense mutation in 1 case and missense mutation in 2 cases. The low‐grade serous carcinoma exhibited wild type P53 expression. P16 block expression was found in high‐grade serous carcinoma in contrast to mottled expression in low‐grade serous carcinoma (Figure 5J). The positive rate of Ki67 in sample 1 was 64.9%, while the positive rate of Ki67 in sample 2 was 20.5% (Figure 5K).

## 4 DISCUSSION

Ovarian cancer has the highest mortality rate among all gynecological cancers because patients are generally diagnosed in an advanced stage with the majority of cases displaying platinum resistant relapses.^3^ According to the statistics of Global Cancer Observatory (GCO, https://gco.iarc.fr/), there are a total of 313,959 patients with ovarian cancer patients and 207,252 cases died from it.

Recently, the function of SnoRNAs has been reported, for example, SNORD89 was identified as a prognostic biomarker and prospective therapeutic in ovarian cancer patients and breast cancer patients.^19^, ^20^ Compared with other types of RNA, the metabolism of snoRNAs is very stable and easy to be detected. Studies had been reported that snoRNAs were detected in cancer tissues, cancer cells, cancer patients' serum,^21^ sputum,^22^ urine,^23^ and so on. Some snoRNAs had been reported to participate the metastasis of ovarian cancer.^24^ However, there is a lack of systematic and comprehensive research on SnoRNAs in ovarian cancer. In our study, we comprehensively analyzed the SnoRNA in patients with ovarian cancer, and screened out 14 prognostic SnoRNAs by Univariate Cox survival analysis (Table 4). Then, prognostic model was constructed by Multivariate Cox survival analysis, and 9 SnoRNAs were included in the prognostic model (Table 5). According to the prognostic model, each ovarian cancer patient has a unique RiskScore, and the heat map of survival time, RiskScore and vital status showed that patients with higher RiskScore had more deaths and lower survival time (Figure 1A,B,E). Moreover, we analyzed the relationship between CA125 and the prognosis of patients with ovarian cancer. However, KM Plot showed that the conventional biomarker CA125 cannot predict the prognosis of ovarian cancer well (Figure S2A).

A good prognostic marker is often associated with multiple clinicopathological parameters. Hence, we analyzed the correlation of the RiskScore with different clinicopathological parameters, including age, tumor size, lymphatic invasion, stage and tumor status of ovarian cancer patients. Results showed that RiskScore was significantly increased in patients with higher age, larger TumorSize, advanced stage and with tumor status (Figure S1A,B,D,E). Also, we analyzed the expression of CA125 in different clinicopathological parameters. Results showed CA125 downregulated in higher age and upregulated in lymphatic invasion patients (Figure S2B,D). There was no difference in expression in other subgroup of clinicopathological parameters (Figure S2C–F). These results suggested that the RiskScore was closely related to the clinical parameters and may have important clinical value.

Some SnoRNAs had been reported to have co‐expressed with their host genes.^16^ In our research, 7 of 9 SnoRNAs in the prognostic model had positive correlation with their host genes in ovarian cancer tissues (Figure 2A–G). The expression abundance of SNORA70J is very low, alike its host gene RPSAP71 (Figure 2G). CNVs has been reported occurred in various cancers, and the expression of some SnoRNAs were associated with their CNVs.^25^ In our research, 5 of 9 SnoRNAs in our prognostic model had correlation with their CNVs (Figure 2H).

To validate the specificity and sensitivity of the prognostic model we constructed, ROC curve was drawn. In different time spans, the area of 7 years achieved 0.785. These results showed that the model has the best effect in predicting the prognosis of 7 years for ovarian cancer patients (Figure 3A). Meanwhile, we detected the ROC of the conventional biomarker CA125. However, CA125 has no good specificity and accuracy in indicating the prognosis of patients with ovarian cancer (Figure S2G).

Moreover, results of Univariate and multivariate Cox survival analysis further confirmed that the RiskScore was an independent prognostic factor in ovarian cancer patients (Figure 3B,C). Stratified analysis of survival according to different clinical parameters was conducted. We found that RiskScore predict prognosis well in diverse ages, TumorSize and stage (Figure 4G–O). However, RiskScore, in tumor free and no lymphatic invasion patients, could not predict patients' prognosis well (Figure 4C,F). We speculated that these results may be caused by the small number of experimental cases.

Moreover, patients with ovarian cancer were randomly divided into two groups, and validate the RiskScore in each subgroup. All of the results showed that patients with high RiskScore had poorer prognosis versus patients with low RiskScore (Figure 5A–H). GEO database was included to verify the prognostic model, and results showed that patient with high RiskScore had poor prognosis (Figure S2H).

Further, we detected the expression of SnoRNAs in 7 paired tissues, and results suggested that all of them, except SNORD3C and SNORD89, down regulated in ovarian cancer tissues compared to ovarian normal tissues (Figure 5I). And, this result is in accord with the previous research.^19^ According to the prognostic model we constructed, the RiskScore of clinical sample 1 and sample 2 are 46.47 and 2.469, and this result indicates that sample 1 had poorer prognosis versus sample 2. Moreover, the results of H&E staining and immunohistochemistry of Ki67, P53 and P16 confirmed that patients with high RiskScore are more malignant. The positive rate of Ki67 in sample 1 was 64.9%, and higher than that 20.5% in sample 2 (Figure 5J,K). And, P16 block expression was found in sample 1 in contrast to mottled expression in sample2 (Figure 5J).

In addition, plate clone formation assay showed that overexpression of snoRD126 and snoRD89 significantly increased the number of clone formation in ovarian cancer cells, and the size was larger in OE group (Figure S2I). And, we found overexpression of snoRD126 and snoRD89 can upregulate the expression of cMyc (Figure S2J). Coincidentally, cMyc is an important factor affecting the stem of various cancer cells, such as neuroblastoma cells,^26^ glioma stem cells,^27^ and so on. Therefore, we speculate that snoRD89 and snoRD126 may affect the prognosis of ovarian cancer by regulating the stem of ovarian cancer cells. However, the detailed molecular mechanism still needs to be further studied.

## 5 CONCLUSIONS

In summary, we identified a nine‐SnoRNAs signature as an independent indicator to predict prognosis of ovarian cancer patients, providing a prospective prognostic biomarker and potential therapeutic targets for ovarian cancer.

## CONFLICT OF INTEREST

The authors declare that they have no competing interests.

## AUTHORS' CONTRIBUTIONS

WJZ designed the experiments. TZ, SHL, QNK and WMH performed the experiments. XZ and WJZ analyzed the experimental data. WH, FBZ and WJZ wrote and reviewed the manuscript. All authors read and approved the final manuscript.

## ETHICS APPROVAL AND CONSENT TO PARTICIPATE

All patients consented to the institutional review board which allows comprehensive analysis of tumor specimens.

## CONSENT FOR PUBLICATION

Not applicable.

## Supporting information


FigureS1

FigureS2

## Data Availability

The datasets analyzed during the current study are available in the TCGA repository, https://cancergeno me.nih.gov/.

## References

[1] Kuroki L , Guntupalli SR . Treatment of epithelial ovarian cancer. BMJ. 2020;371:m3773. 33168565 10.1136/bmj.m3773

[2] Siegel RL , Miller KD , Jemal A . Cancer statistics, 2020. CA Cancer J Clin. 2020;70(1):7‐30. 31912902 10.3322/caac.21590

[3] Lheureux S , Braunstein M , Oza AM . Epithelial ovarian cancer: evolution of management in the era of precision medicine. CA Cancer J Clin. 2019;69(4):280‐304. 31099893 10.3322/caac.21559

[4] Alkema NG , Wisman GBA , van der Zee AGJ , van Vugt MATM , de Jong S . Studying platinum sensitivity and resistance in high‐grade serous ovarian cancer: different models for different questions. Drug Resist Updat. 2016;24:55‐69. 26830315 10.1016/j.drup.2015.11.005

[5] Christie EL , Bowtell DDL . Acquired chemotherapy resistance in ovarian cancer. Ann Oncol. 2017;28(suppl_8):viii13‐viii15. 29232469 10.1093/annonc/mdx446

[6] Radu MR , Prădatu A , Duică F , et al. Ovarian cancer: biomarkers and targeted therapy. Biomedicines. 2021;9(6):693‐714. 34207450 10.3390/biomedicines9060693 PMC8235073

[7] Liang J , Wen J , Huang Z , Chen XP , Zhang BX , Chu L . Small nucleolar RNAs: insight into their function in cancer. Front Oncol. 2019;9:587. 31338327 10.3389/fonc.2019.00587 PMC6629867

[8] Liu Y , Ruan H , Li S , et al. The genetic and pharmacogenomic landscape of snoRNAs in human cancer. Mol Cancer. 2020;19(1):108. 32576192 10.1186/s12943-020-01228-z PMC7313177

[9] Lemus‐Diaz N , Ferreira RR , Bohnsack KE , Gruber J , Bohnsack MT . The human box C/D snoRNA U3 is a miRNA source and miR‐U3 regulates expression of sortin nexin 27. Nucleic Acids Res. 2020;48(14):8074‐8089. 32609813 10.1093/nar/gkaa549 PMC7430653

[10] Zhou F , Liu Y , Rohde C , et al. AML1‐ETO requires enhanced C/D box snoRNA/RNP formation to induce self‐renewal and leukaemia. Nat Cell Biol. 2017;19(7):844‐855. 28650479 10.1038/ncb3563

[11] McCann KL , Kavari SL , Burkholder AB , Phillips BT , Hall TMT . H/ACA snoRNA levels are regulated during stem cell differentiation. Nucleic Acids Res. 2020;48(15):8686‐8703. 32710630 10.1093/nar/gkaa612 PMC7470967

[12] Bratkovič T , Božič J , Rogelj B . Functional diversity of small nucleolar RNAs. Nucleic Acids Res. 2020;48(4):1627‐1651. 31828325 10.1093/nar/gkz1140 PMC7038934

[13] Xu M , Chen X , Lin K , et al. lncRNA SNHG6 regulates EZH2 expression by sponging miR‐26a/b and miR‐214 in colorectal cancer. J Hematol Oncol. 2019;12(1):3. 30626446 10.1186/s13045-018-0690-5 PMC6327409

[14] Shan P , Yang F , Qi H , et al. Alteration of MDM2 by the small molecule YF438 exerts antitumor effects in triple‐negative breast cancer. Cancer Res. 2021;81(15):4027‐4040. 33985974 10.1158/0008-5472.CAN-20-0922

[15] Yoshida K , Toden S , Weng W , et al. SNORA21‐ an oncogenic small nucleolar RNA, with a prognostic biomarker potential in human colorectal cancer. EBioMedicine. 2017;22:68‐77. 28734806 10.1016/j.ebiom.2017.07.009 PMC5552212

[16] Williams GT , Farzaneh F . Are snoRNAs and snoRNA host genes new players in cancer? Nat Rev Cancer. 2012;12(2):84‐88. 22257949 10.1038/nrc3195

[17] Gong J , Li Y , Liu CJ , et al. A Pan‐cancer analysis of the expression and clinical relevance of small nucleolar RNAs in human cancer. Cell Rep. 2017;21(7):1968‐1981. 29141226 10.1016/j.celrep.2017.10.070

[18] Hermens M , van Altena AM , van der Aa M , et al. Ovarian cancer prognosis in women with endometriosis: a retrospective nationwide cohort study of 32,419 women. Am J Obstet Gynecol. 2021;224(3):284.e1‐284.e10. 32841629 10.1016/j.ajog.2020.08.056

[19] Zhu W , Niu J , He M , et al. SNORD89 promotes stemness phenotype of ovarian cancer cells by regulating Notch1‐c‐Myc pathway. J Transl Med. 2019;17(1):259. 31395064 10.1186/s12967-019-2005-1 PMC6686521

[20] Krishnan P , Ghosh S , Wang B , et al. Profiling of small nucleolar RNAs by next generation sequencing: potential new players for breast cancer prognosis. PLoS One. 2016;11(9):e0162622. 27631501 10.1371/journal.pone.0162622 PMC5025248

[21] Appaiah HN , Goswami CP , Mina LA , et al. Persistent upregulation of U6:SNORD44 small RNA ratio in the serum of breast cancer patients. Breast Cancer Res. 2011;13(5):R86. 21914171 10.1186/bcr2943 PMC3262198

[22] Su Y , Guarnera MA , Fang HB , Jiang F . Small non‐coding RNA biomarkers in sputum for lung cancer diagnosis. Mol Cancer. 2016;15(1):36. 27176474 10.1186/s12943-016-0520-8 PMC4866414

[23] Egidi MG , Cochetti G , Guelfi G , et al. Stability assessment of candidate reference genes in urine sediment of prostate cancer patients for miRNA applications. Dis Markers. 2015;2015:973597. 26078486 10.1155/2015/973597 PMC4452852

[24] Yang Y , Zhang H , Xie Y , et al. Preliminary screening and identification of differentially expressed metastasis‐related ncRNAs in ovarian cancer. Oncol Lett. 2018;15(1):368‐374. 29387224 10.3892/ol.2017.7338 PMC5769367

[25] Zhao Y , Yan Y , Ma R , et al. Expression signature of six‐snoRNA serves as novel non‐invasive biomarker for diagnosis and prognosis prediction of renal clear cell carcinoma. J Cell Mol Med. 2020;24(3):2215‐2228. 31943775 10.1111/jcmm.14886 PMC7011154

[26] Le Grand M , Mukha A , Püschel J , et al. Interplay between MycN and c‐Myc regulates radioresistance and cancer stem cell phenotype in neuroblastoma upon glutamine deprivation. Theranostics. 2020;10(14):6411‐6429. 32483461 10.7150/thno.42602 PMC7255021

[27] Fukasawa K , Kadota T , Horie T , et al. CDK8 maintains stemness and tumorigenicity of glioma stem cells by regulating the c‐MYC pathway. Oncogene. 2021;40(15):2803‐2815. 33727660 10.1038/s41388-021-01745-1

